# Subpopulations of Addictive Behaviors in Different Sample Types and Their Relationships with Gender, Personality, and Well-Being: Latent Profile vs. Latent Class Analysis

**DOI:** 10.3390/ijerph18168590

**Published:** 2021-08-14

**Authors:** Paweł A. Atroszko, Bartosz Atroszko, Edyta Charzyńska

**Affiliations:** 1 Faculty of Social Sciences, University of Gdańsk, 80-309 Gdańsk, Poland; p.atroszko@ug.edu.pl (P.A.A.); bartosz.atroszko@gmail.com (B.A.); 2 Faculty of Social Sciences, University of Silesia in Katowice, 40-007 Katowice, Poland

**Keywords:** behavioral addictions, co-occurrence, latent profile analysis, latent class analysis, personality, prevalence, well-being

## Abstract

Background: Relatively strong theoretical assumptions and previous studies concerning co-occurring addictive behaviors suggest a subpopulation representing general proclivity to behavioral addictions (BAs), and there are gender-specific subpopulations. This study aimed to compare latent profile analysis (LPA) and latent class analysis (LCA) as the methods of investigating different clusters of BAs in the general student population and among students positively screened for at least one BA. Participants and procedure: Analyses of six BAs (study, shopping, gaming, Facebook, pornography, and food) and their potential antecedents (personality) and consequences (well-being) were conducted on a full sample of Polish undergraduate students (*N* = 1182) and a subsample (*n* = 327) of students including individuals fulfilling cutoff for at least one BA. Results: LPA on the subsample mostly replicated the previous four profiles found in the full sample. However, LCA on a full sample did not replicate previous findings using LPA and showed only two classes: those with relatively high probabilities on all BAs and low probabilities. LCA on the subsample conflated profiles identified with LPA and classes found with LCA in the full sample. Conclusions: LCA on dichotomized scores (screened positively vs. negatively) were less effective in identifying clear patterns of interrelationships between BAs based on relatively strong theoretical assumptions and found in previous research. BAs can be investigated on the whole spectrum of behavior, and person-centered analyses might be more useful when they are based on continuous scores. This paper provides more detailed analyses of the four basic clusters of BAs, prevalence, and co-occurrence of particular BAs within and between them, their gender and personality risk factors, relationships to well-being, and their interrelationships as emerging from the results of this and previous studies.

## 1. Introduction

A recent study using latent profile analysis (LPA) showed four profiles of behavioral addictions (BAs) severity, including a profile with a general proclivity to addiction, two gender-specific profiles, one almost exclusively female and a second predominantly male, and a profile with low levels of BAs [1]. These profiles showed different relationships with Big Five personality traits and narcissism and with a wide range of well-being indicators. The results are summarized in Table 1. The aim of this study was to investigate profiles of BAs among students screened for at least one BA and their similarities to patterns in the general student population. Furthermore, comparisons of results using LPA and LCA with dichotomized scores (screened positively for at least one BA vs. screened negatively) can provide valuable information on the relative usefulness of these two person-centered methods for investigating BAs and substance use disorders (SUDs), constituting two major classes of addictive behaviors. This is particularly important as LCA, not infrequently performed on dichotomous scores, is still the predominant method to investigate addictive behaviors within a person-centered approach (see Table 2). However, based on the limited information used in such analyses, it can be expected that it may not be the optimal approach to investigating clusters of addictive behaviors. We address it in detail in the paper. Additionally, the analysis of prevalence and co-occurrence patterns of BAs across profiles can provide more new in-depth knowledge on the nature of the profiles and particular BAs, which will further emphasize their meaning in the context of the proper analytical approach from which they are derived. Especially, gender differences in BAs are apparent but poorly understood. This paper provides more detailed analyses of gender and personality risk factors and their interrelationships as emerging from the results of this and previous studies. To the Authors’ knowledge, there are no studies so far comparing LPA and LCA conducted in a sample representing the general student population and among students screened for at least one addictive behavior. While certain differences may be predicted based on the nature of these analyses (e.g., effects of reduced information in LCA in comparison with LPA), an illustrative example of real-life data may more compellingly emphasize these effects and guide future practice. Moreover, the analyses of differences in potential antecedents and consequences of clusters and of differences in prevalence rates of BAs within clusters obtained with different analytical methods may more clearly show the importance of the methodological approach to the study of patterns of co-occurrences of addictive behaviors.

## 2. Behavioral Addictions, Latent Trait Model, and Person-Centered Approach

Substantial co-occurrence among different addictive behaviors and pronounced gender differences in the risk for particular addictions and clusters of addictions [2,3,4] suggest that the addictive process may be better understood if these patterns of interrelationships are taken into account. General proclivity for addictive behaviors can be seen among susceptible individuals and is related to the conceptualization of addiction as one underlying process with different manifestations [5,6]. The prevalent approach concentrates on single addictive behaviors. It foremostly uses the latent trait model, which conceptualizes addiction as a latent construct that can be measured along a dimension of severity [7]. This variable-centered approach assumes that people in a particular sample are drawn from a single homogeneous population. Consequently, the relationships between variables are treated as identical across all individuals. Alternative approaches to psychopathology include the person-centered approach, which was gradually introduced to addiction research [8,9,10]. It assumes that different subpopulations or groups representing various symptoms or disorders, including their risks, can be identified and studied [9]. This approach is not mutually exclusive with the latent trait approach, and when guided by proper research questions, it provides a different type of potentially useful information in understanding psychopathology. One of the most important advantages of the person-centered approach is that it allows investigating the changes in addiction with time and its potential antecedents and consequences in different groups of addicted individuals.

Latent trait modeling and person-centered approaches can, to a large extent, give similar results, particularly in situations where profiles or classes of symptoms of a single addictive behavior are distinguished but differ only in the severity of symptom clusters (e.g., [11]). In fact, such a situation is sometimes used to establish the psychometric properties of a diagnostic measure, particularly its specificity and sensitivity (e.g., [12]). Different clustering may be revealed when additional variables are added to the model (apart from the general score on a single addictive behavior or its symptoms). These additional variables often include personality or psychopathology [13]. However, in many cases, these clusters still mainly distinguish the severity of addiction [14]. A specific case is a model with numerous different addictive behaviors. These models also frequently find clusters representing addiction risk or severity [15]; however, they sometimes provide more complex co-occurrence patterns [16]. Furthermore, clusters based on potential antecedents or risk factors have also been investigated [17,18]. In such cases, the distinguished groups reflect common etiology and can then be related to patterns of common symptomatology (co-occurring addictive behaviors). Finally, latent transition analysis allows for identifying different development trajectories of addictive behaviors in longitudinal designs [19,20,21]. Moreover, latent class growth analysis integrates person-centered and variable-centered analyses, allowing investigation of the diverse groups of individuals and heterogenous developmental trajectories [22], and it can be effectively used in addiction research [23].

Different factors such as samples (general population vs. clinical) or measurement scales (continuous scores vs. dichotomized) may affect the results of person-centered analyses, particularly by determining the type of possible analysis (latent class or latent profiles). At present, a limited number of studies have compared different person-centered approaches in the addiction field, and therefore, systematic analyses of their properties and performance are lacking. Moreover, LCA is predominantly used, with very few studies applying LPA, and studies analyzing models with numerous addictive behaviors are still very rare. Table 2 presents an overview of notable studies in recent years, including analyses conducted on multiple addictive behaviors. There is considerable heterogeneity among them concerning samples, measures used, types of analyses, and, importantly, results. Most consistently, these yield classes differentiating levels of risk (e.g., [15]) and some gender differences (e.g., [16]), as well as more complex patterns involving both and other specific clustering factors (e.g., [24]); however, very few generalizations can be drawn beyond that.

The categorical classifications, especially dichotomous (e.g., addicted vs. non-addicted), may be attractive due to easy and intuitive interpretation, as well as simple and convenient measurement. However, it should be emphasized that proper justification for the choice of categorical measurement of addictive behaviors or for recoding continuous scores into a categorical variable is typically missing in the papers using such an approach (see papers in Table 2). It is not surprising because, generally, there is little validity to measuring addictive behaviors or addiction risk as a dichotomous or categorical variable in most settings. Only in very unusual situations could such categorizations be performed validly. For example, in relation to only one addictive disorder, there are extreme, almost pure cases of alcohol addiction, e.g., individuals with a drinking problem of several decades and Korsakoff syndrome. There are also pure cases of non-addicted individuals, such as people who do not drink alcohol at all. However, there is an overwhelming majority of cases of different levels of addiction risk between these extremes, which can be modeled and investigated with continuous scores on valid and reliable measures.

Furthermore, classifying only those in treatment as addicted (in situations in which they are compared with a non-addicted group) is highly problematic since the vast majority of addicted individuals never seek help, and there is considerable heterogeneity among treated individuals. In such cases, being in treatment should be considered a separate variable from an individual’s addiction status. Additionally, it may be appropriate to analyze, for example, a set of symptoms (present vs. absent) of alcohol addiction with LCA and find various classes [22]. However, classifying individuals as addicted vs. non-addicted is a different situation that is highly controversial and, in most cases, problematic. It may be a subtle difference for some researchers, but it is evident and has significant consequences for analyzing addictive behaviors. Moreover, even particular symptoms may be measured along a continuum of severity, which is a preferable option to dichotomous categorization (present vs. absent). A recent study showed that latent profile analysis applied to BAs in a general student sample could distinguish clear and distinct clusters representing general proclivity to addiction and gender-specific profiles [1]. This not only allows one to identify groups of individuals with different severity of addiction but also to identify qualitatively distinctive profiles representing gender-specific addictive behaviors. Such a property of the analytical approach can be very useful in systematically investigating antecedents, consequences, course, prognosis, and treatment response of these different subpopulations represented by profiles.

## 3. General Proclivity for Addictions

Addictions share common etiological factors and expressions, i.e., manifestations and sequels, which can be organized in biological, psychological, and social clusters; natural history; treatment non-specificity; and object substitution [5,6]. These suggest one underlying addiction syndrome with multiple opportunistic expressions. Typically, these expressions are conceptualized in terms of unique manifestations, such as alcohol drinking, gambling, smoking, etc. However, the addiction syndrome model also implies something that could be denoted with the term “general proclivity for addiction”. It describes individuals with the highest risk for such a syndrome and numerous simultaneous expressions of different addictive behaviors [1,6]. In most extreme and perhaps clear cases, individuals with such proclivity will show, at least to some extent, problematic behaviors in relation to most of the addictive behaviors [15,16,29]. Common etiological factors include genetic risks; psychosocial factors, including personality risks (emotional instability, low conscientiousness and agreeableness, and high narcissism and impulsivity [16,18,35,36]); environmental factors, including familial risks (e.g., socioeconomic hardship or neglect); other individual (e.g., early trauma and/or mental problems) risk factors; and their interactions.

From this perspective, addiction syndrome may also manifest in specific clusters of co-occurring addictive behaviors with their particular specific risk factors, apart from generally shared etiology. One such crucial factor is gender, which differentiates risks not only for SUDs [2] but also shows clear patterns in relation to BAs [1]. In the latter case, the differences in gender risks are currently poorly understood. General proclivity for addiction seems to be related to the male gender, as males typically show higher rates of SUDs than females [28,37].

## 4. Gender and Addiction: Different Profiles for Women and Men

The gender differences concerning the risk of addiction and various types of addictive behaviors are well-established [2,3,4,38] but noticeably less understood in relation to BAs in comparison with SUDs [1,16,37,39]. Differences in emotional sensitivity to stress-system changes between women and men are crucial in explaining sex differences in SUDs [40]. The higher risks for SUDs among men are explained by their higher risk-taking and stimulation-seeking behavior and their impulsiveness compared with women. In other words, men more often use substances to enhance their positive mood. Women tend to use substances as coping resources to alleviate negative emotions [39].

There is a similar general rationale referring to emotional sensitivity to stress-system changes, related to different motives for performing particular behaviors (stimulation seeking vs. negative emotion avoidance), most often grounded in evolutionary psychology and sex roles related to hunter–gatherer societies. This reasoning is explicitly or implicitly involved in providing theoretical explanations for gender differences in BAs. Most data come from studies on particular BAs and provide some idiosyncratic theoretical reasons, discussed below, for the observed differences. Previous studies showed that study [41,42], social networking [43], Facebook [44], shopping [45], and food [46] addictions are more prevalent among women. On the other hand, men constitute most of the population addicted to gaming [47] and pornography [48]. Additionally, some studies using a person-centered approach identify gender-specific profiles with predominant shopping or eating addiction among women and sex addiction among men [28].

Shopping addiction has been explained in terms of evolutionary psychology, referring to collecting tendencies that had been typically assigned to females within their social groups [49]. Internet gaming disorder has been explained from an evolutionary perspective suggesting that males are more aggressive and competitive in maintaining dominance and defending territory, while aggressiveness is not typically expected socially from females in most cultures [43]. On the other hand, social networking sites addiction is more prevalent among women because of females’ sensitivity to social signals and the valuation of interpersonal relations [43]. Sex-specific hormonal differences related to the menstrual cycle are typically identified as underlying gender differences in food addiction [50,51]. Again, pornography consumption and addiction can be explained by evolutionary short-term mating preferences, indicating that men are typically more responsive to mating opportunities with healthy women in reproductive age [52,53,54,55]. Within this framework, study addiction could be arguably linked to female sensitivity to social signals and valuation of interpersonal relationships, as academic failure is related to social exclusion and marginalization [56]. It is consistent with studies showing the relationship of study addiction to prosocial values [41]. Generally, evolutionary-oriented researchers suggest that sex/gender differences observed in the brain and reflected in behavior may be associated with distinct demands for female childcare and male hunting activities [57]. However, sociocultural factors, including socioeconomic or gender role conformity, are typically suggested as important moderators of these tendencies. In comparison with SUDs, more complex mechanisms involving biological and social factors are probably accounting for the sex and gender differences in BAs [3]. While evolutionary explanations have the advantage of generality and simplicity, a more nuanced approach to identifying immediate factors and biological underpinnings determining different BAs risks is necessary. Women are more punishment sensitive [58], may be susceptible to social expectations, particularly in relation to public appearance (e.g., academic performance, social status), and react to stress and cope with it differently than men [40]. Identifying different profiles of co-occurrence of potential BAs may significantly help to investigate these probable causes of sex and gender differences in BAs.

## 5. Personality and Addiction

Previously, the profile representing general proclivity to BAs was related to the highest narcissism, and lowest emotional stability among all profiles ([1]; see Table 1). Similar to the male-majority profile (high pornography and gaming), it showed the lowest conscientiousness among all profiles. This is congruent with other studies on particular addictive behaviors [16,18,35,36,59]. However, the female-majority profile showed the highest conscientiousness and extraversion among all profiles, including the one with medium–low levels of all BAs, unlike in some previous research on study, Facebook, shopping, and food addictions [42,44,60,61,62]. This finding suggests a few possible explanations, such as the female-majority profile (i) constitutes an early phase on a trajectory to a full-blown addiction syndrome, and/or (ii) represents one of the different types of study, Facebook, shopping, and food addictions characteristic of women. For example, it has been repeatedly shown that shopping addiction has at least two major subtypes, including a neurotic with low self-esteem type (consistent with self-medication) and impulsive (consistent with sensation-seeking type) [14]. Similarly, different types have been proposed for work addiction, including compulsive perfectionistic/controlling and impulsive/hyperactive [63,64,65], and study addiction, including socially conforming (mostly women) and narcissistic/grandiose (mostly men) [1,66]. Importantly, study addiction was consistently positively related to conscientiousness and deteriorated psychosocial functioning in the previous studies in different countries, among undergraduate and high school students, and among women and men [41,42,67,68]. These findings suggest that conscientiousness may, in some cases, not be a protective factor against addiction and ill-health [69], particularly by affecting work-related behaviors [70]. Chronic job- or study-related stress may, in turn, be associated with higher risks of other compulsive behaviors related to food intake [71], shopping [72], and social networking [73]. These findings strongly support the notion that, in addition to common personality risk factors (such as neuroticism), there could be specific risk factors not only for particular addictions (e.g., high conscientiousness in studying) but also for profiles of addictions, such as the female-majority profile.

A person-centered approach to investigating addiction may be indispensable because otherwise, results such as different types of particular addictions and within-addiction variability of risk factors might be ambiguous and confusing. Reviews of findings of studies based on a person-centered approach showed that this methodology might produce significant benefits and more accurate models of vulnerability to psychiatric disorders [74,75]. Some studies of BAs already confirm this assumption [1,14]. However, the development of proper standards for these analyses is highly warranted; otherwise, even the results of person-centered analyses may be heterogenous and difficult to interpret and generalize [9,76].

## 6. Prevalence and Co-Occurrence

The analyses of prevalence and co-occurrence of BAs within and between properly identified clusters may allow for a better understanding of the addictive process and of the specificity and importance of particular addictive behaviors. Since LCA and LPA may produce different results, it has a significant meaning for the way we understand the co-occurrence of BAs, its causes, and consequences. Gaming disorder and gambling disorder are classified as disorders due to addictive behaviors in the eleventh revision of the International Classification of Diseases [77]. Nevertheless, no diagnostic gold standard for most BAs has been established, and even for the BAs with officially recognized diagnostic symptoms, the estimates of prevalence vary significantly. The choice of screening tool may account for most of the variance [78]. Furthermore, very infrequently, data from representative samples of general populations are available. The Supplementary Materials contain a short overview of the prevalence studies. There, we provide the best available data on the prevalence of BAs in the general population based on representative samples against the backdrop of varying estimates coming from convenience samples. This provides a wider context for the interpretation of the results of the current study, particularly the relative proportions of different BAs prevalences and the amount of overestimation of prevalence with convenience samples in comparison with nationally representative samples.

The pooled prevalence of Internet gaming disorder (IGD) among adolescents in 16 studies was 4.6% [47], similar to 4.7% in the general population [79,80,81]. However, data from nationally representative samples suggest that the prevalence is considerably lower in the general population (only 0.7% to 1.4% of the gamers population [82,83], which in turn constitutes only a portion of the general population). The prevalence estimates of social networking sites (SNS) addiction range from 5% to 25% [84]. The surveys on representative samples showed that 4.5% of persons belonged to the at-risk group for problematic social media use in an adolescents’ sample in Hungary [85], and 2.9% compulsively used social networking sites in an adult sample in Belgium [86]. The prevalence of social media addiction is typically higher than Facebook addiction, as measured in the same sample, and highly depends on the country [84,87,88,89]. The estimates of the prevalence of compulsive buying in representative studies range from 1% to 8.1%, with a mean pooled estimate of 4.9% [12]. Food addiction prevalence estimates range from 2.6% among Dutch adolescents [90] to 56.8% among obese patients with binge eating disorder [91]. In a nationally representative sample in Germany, the prevalence was 7.9% [92]. Researchers seem to be most reluctant to provide problematic pornography estimates [93,94], probably due to the highly controversial nature of this behavior and its investigation [95]. Finally, study addiction is conceptualized as a potential early form of work addiction [41,67] and was related to it in a longitudinal study [96]. Study addiction, as well as work addiction, are consistently found to have similar prevalence rates and to be highest among all BAs [42,97,98,99], comparable maybe only with food addiction [46,92]. In Norway, a nationally representative survey among employees showed an 8.3% prevalence of work addiction [100], which was very similar to the prevalence of study addiction (9.7%) among undergraduate students [41]. In Poland, rates varied from 6.4% to 14.2–16.0% [41,97], and prevalence was 15.4% among high school students [42]. These are again very similar to the estimates of the prevalence of work addiction in Poland (17.4%) [101]. In India, study addiction was found among 17.1% of high school students [102].

It needs to be emphasized that there are currently no reliable estimates of BAs comorbidity [99] due to (i) no gold standard diagnostics for BAs, (ii) arbitrary and varying cutoff scores based on different psychometric tools for most BAs, (iii) arbitrary choice of a particular set of addictions in studies (see for example [18,103]), or (iv) not reporting co-occurrences in multiple BAs studies [24]. The most often co-occurring addictions seem to be those related to similar (i) medium, e.g., Internet, including Facebook and gaming addiction [18,103]; (ii) behavior, such as love, sex, pornography [99]; (iii) risk factors, such as rigid perfectionism (food addiction and exercise dependence in amateur endurance athletes [104]); or (iv) mechanisms of dysregulation, e.g., ingesting mood-altering foods or substances (symptoms of food addiction positively associated with alcohol use, cannabis use, and smoking in a group of Dutch adolescents [90]). As a result of this complexity, at present, it is almost impossible to draw conclusions related to general patterns of comorbidities among a wide range of BAs. However, based on the previous research and theoretical models, it can be expected that gender-specific BAs, which may represent higher-order constellations of risk factors and dysregulation mechanisms, may co-occur more often [1].

## 7. Hypotheses

Relatively strong theoretical assumptions concerning co-occurring addictive behaviors suggest that there is a cluster reflecting general proclivity to behavioral addictions (BAs) and that there are gender-specific clusters. These are also supported by previous studies. However, the differences between LPA and LCA, particularly regarding the reduced information used by LCA, suggest that these methods may produce different profiles/classes and that LCA may be less effective in identifying clear patterns of interrelationships between BAs than LPA. Following the assumptions behind the confirmatory approach in science [105], the predictions are not based on technical differences in the analytical method but on the expectations based on substantive processes related to addictive behaviors. Against this backdrop, the performance of both methods in identifying theoretically expected patterns can be investigated. Similarly, range restriction effects may occur in the subsample and affect LPA. Therefore, it was hypothesized that (i) LPA on a subsample of students screened for at least one BA will, to a large extent replicate, findings with LPA on a general student sample (H1); (ii) LCA in the general student sample will produce similar results to LPA (H2); (iii) LCA in a subsample will produce similar results to LCA in the general student sample and results of LPA in both samples (H3); and (iv) patterns of potential antecedents and consequences will be similar across samples and methods (H4).

## 8. Methods

### 8.1. Sample and Procedure

The general sample of students consisted of 1182 participants (25 responses were eliminated due to more than 70% of missing data) and was described in detail elsewhere [1]. All participants were undergraduate students who were studying at universities in Gdańsk: University of Gdańsk, Gdańsk Technological University, and Gdańsk University of Sport and Recreation.

The subsample was extracted from the full sample. To be included in the current sample, a person had to meet the criteria for at least one BA. To be classified as a person meeting the criteria for a BA, participants had to score four or five points for at least four diagnostic criteria (see the detailed description of cutoff scores in [1]). The final sample in the current study consisted of 327 persons: 190 women (58.1%) and 132 men (40.4%); five participants (1.5%) did not indicate their gender. The mean age of the participants was 20.55 years (SD = 1.66). Students were affiliated with different faculties, courses of study, and years of study.

Data collection used convenience sampling. The study was a part of a larger project, “Study addiction in the context of other behavioral addictions: Co-occurrence, potential determinants and consequences from the perspective of the addiction model as an ineffective way of coping with stress”. Data collection took place during the winter semester (October–December) in 2016. It finished several weeks before the examination session to reduce the effects of exam stress. This was particularly important in relation to such variables as study addiction. BAs questionnaires asked about experiences from the previous year (12 months). Lecturers were first contacted via e-mail to invite their students to the study. Then, students were invited to participate anonymously in the study during lectures or classes. The estimated response rate was above 95%. No monetary or other material rewards were offered. Filling in the paper-and-pencil questionnaires took on average 15–20 min.

### 8.2. Measures

Table 3 presents the main characteristics of the instruments used in the study. More details can be found elsewhere [1]. Six measures of behavioral addictions, two of personality, and four well-being measures were administered, along with demographics.

## 9. Statistical Analysis

In the preliminary analysis, we calculated descriptive statistics and zero-order correlations. Next, we conducted comparisons between the persons without BAs and persons with at least one BA in terms of sociodemographics, personality, and well-being indicators. Then we examined what percentage of participants met the cutoff of a particular BA and the co-occurring BAs.

In the next step of the analysis, we performed three analyses using a person-centered approach: (i) latent profile analysis for BAs treated as continuous variables, carried out among persons with at least one BA (*n* = 327); (ii) latent class analysis for BAs treated as nominal variables (0 = the cutoff not reached; 1 = the cutoff reached), conducted in the full sample (*n* = 1157); (iii) latent class analysis for BAs treated as nominal variables (0 = the cutoff not reached; 1 = cutoff reached), carried out among persons with at least one BA (*n* = 327). For each analysis, we first examined models containing from one to seven clusters and compared them using the model fit criteria recommended by the simulation studies [115,116]: the Bayesian information criterion (BIC), consistent Akaike information criterion (CAIC), and sample-size adjusted BIC (SABIC). Better model fit is indicated by the lower values of BIC, CAIC, and SABIC. To provide meaningful solutions, we also considered the theoretical interpretability of the clusters, model parsimony (achieving an acceptable model fit with the minimum number of groups), and the size of the smallest group (groups smaller than 5% of the sample usually considered spurious and unreplicable [117,118,119,120]). In addition, we calculated the entropy value (entropy *R*^2^ [121]), which indicates how well the profile/class memberships can be predicted from the observed responses. Higher values of entropy indicate better class/profile membership prediction [120]. We further inspected the classification uncertainty by calculating the average posterior probabilities and the expected misclassification rate [120,122]. An average posterior probability value of at least 0.70 indicates well-separated clusters [123].

After establishing the optimal number of clusters, we calculated bivariate residuals (BVRs) to test the assumption of local independence. BVRs greater than 3.84 identify correlations between the pairs of indicators that the model has not adequately explained at α = 0.05 [124]. In the case of large BVRs, we added corresponding direct effect(s) (i.e., the residual associations between pair(s) of indicators) to the current model [125].

When the optimal number of clusters was established, participants were classified into clusters using their probability scores. Using the submodule Step3 included in Latent GOLD 5.1 [121], we tested the potential predictors and consequences of latent profile/class membership. To make the results comparable across the analyses and the samples, the external variables included in each analysis were the same as in the previous paper [1]. Specifically, potential predictors included sociodemographics (gender and age) and personality traits (Big Five personality and narcissism), and potential consequences comprised several well-being indicators: the general quality of life, health quality, sleep quality, perceived stress, anxiety, and hopelessness. The maximum likelihood (ML)-based correction method [126] was used to examine the significance of potential predictors, whereas the Bolck, Croons, and Hagenaars (BCH) correction method [127] was applied to test mean differences across the clusters. Latent GOLD version 5.1 [121] and IBM SPSS Statistics version 26 (IBM Corp. Released 2019; Armonk, NY, USA) were used to perform all calculations.

## 10. Results

### 10.1. Preliminary Analysis

#### 10.1.1. Descriptive Statistics and Correlations

Table 4 presents the descriptive statistics and correlations between the study variables. For potential BAs, the results are shown both for continuous (i.e., level of potential BAs) and categorical (being positively screened for BAs or not) variables.

Table S1 shows differences between persons without BAs and persons with at least one BA in terms of sociodemographics, personality, and well-being indicators. Compared with the former group, the latter had higher levels of all BAs, was older, scored higher on narcissism, perceived stress, anxiety, and hopelessness, and lower on agreeableness, emotional stability, the general quality of life, health quality, and sleep quality. These results substantiate the validity of the used cutoff scores.

#### 10.1.2. Prevalence and Co-Occurrence of BAs

Almost half of the participants (*n* = 161; 49.2%) met the criteria for study addiction (see Table 4). The next most common addiction was food addiction (33.9%), followed by gaming addiction (19.0%), Facebook addiction (18.3%), shopping addiction (12.5%), and pornography addiction (6.4%).

As shown in Table 5, participants who fulfilled the cutoff for pornography addiction were the most likely to have the co-occurring BA(s) (on average 27.6% of them), followed by participants who fulfilled the criteria for shopping addiction (on average 25.4% of them). Furthermore, food addiction and study addiction were most prevalent among individuals with other BAs (on average, 32.4% and 29.4% of all cases, respectively).

### 10.2. Latent Profile Analysis (Students Positively Screened for at Least One BA)

#### 10.2.1. Latent Profiles

**Figure 1:**
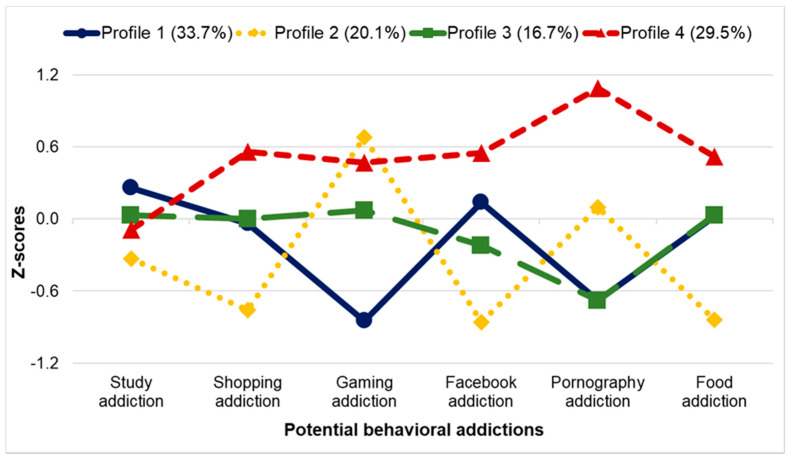
Four-Profile LPA Model Results. Values of all indicators were standardized. *N* = 327.

The procedures for identifying the model best fitting to the data are described in Supplementary Materials, and the results of the comparison of latent profile models are presented in Table S2 and Figure S1. Figure 1 presents the final four-profile solution established by LPA. To make the interpretation and comparisons easier, the established latent profiles were re-ordered to have numbers corresponding to the latent profiles identified in the full sample [1]. Profile 1 (33.7%) grouped the participants with elevated levels of study, Facebook, shopping, and food addictions. Members of Profile 2 (20.1%) had elevated levels of gaming addiction and pornography addiction. Profile 3 (16.7%) had average or lower than average levels of all BAs. Profile 4 (29.5%) had elevated levels of most potential BAs. Comparisons of the levels of potential BAs across the four profiles are shown in Table S3.

#### 10.2.2. Latent Profile Membership and External Variables

The relationships between latent profile membership and external variables (i.e., potential predictors and consequences) are presented in Table 6. Compared with the profile with average or lower than average levels of all BAs (Profile 3; 77.7% women), the proportion of women to men was lower in the profile with elevated levels of gaming addiction and pornography addiction (Profile 2; 22.9% women) and the profile with elevated levels of most BAs (Profile 4; 29.6% women) but higher in the profile with elevated levels of study, Facebook, shopping, and food addictions (Profile 1; 96.3% women). Members of the male-majority profile (Profile 2) were younger than members of the female-majority profile (Profile 1) and the general proclivity profile (Profile 4). Moreover, students in the female-majority profile (Profile 1) were more conscientious than students in the profile with average or lower than average levels of all BAs (Profile 3) and the general proclivity profile (Profile 4; see Table 6).

Regarding well-being indicators, the general proclivity profile (Profile 4) demonstrated lower health quality and higher anxiety and hopelessness than students in the female-majority (Profile 1) and male-majority profiles (Profile 2; see Table 6). Moreover, students in the male-majority profile (Profile 2) showed lower anxiety than students in the profile with average or lower than average levels of all BAs (Profile 3).

#### 10.2.3. Prevalence and Co-Occurrence of BAs in Latent Profiles

Table S4 presents the prevalence and co-occurrence of BAs for each profile. The average prevalence of potential BAs was the highest for members of the general proclivity profile (Profile 4; 30.0%), followed by the female-majority profile (Profile 1; 22.0%). Members of the general proclivity profile (Profile 4) were the most likely to fulfill the criteria for co-occurring BAs (on average, 71.8% of members of this profile). Consistent with this, students in this profile were the most likely to have two or more BAs (47.3%; see Table S5).

### 10.3. Latent Class Analysis (General Student Population)

#### 10.3.1. Latent Classes

**Figure 2:**
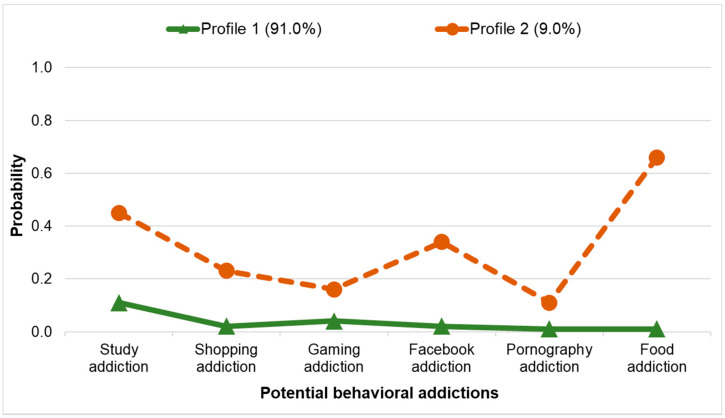
Two-Class LCA Model in the Full Sample. *N* = 1157.

For the LCA in the general student population, the values of model fit criteria consistently suggested that the two-class solution fit the data best (see Table S6). Profile 1 (91.0%) consisted of people with a low probability of potential BAs (see Figure 2 and Table S7). Members of Profile 2 (9.0%) had a heightened probability of all BAs.

#### 10.3.2. Latent Class Membership and External Variables

Students who belonged to the class with a heightened probability of all potential BAs (Class 2) had a higher proportion of women to men (62.2%) compared with students who belonged to the class with a low probability of all potential BAs (Class 1; 51.4%; see Table 7). Moreover, members of Class 2 were older, less emotionally stable, and more narcissistic than members of Class 1. Regarding well-being indicators, members of Class 2 scored lower on general quality of life, health quality, and sleep quality, and they scored higher on perceived stress, anxiety, and hopelessness (Table 7).

### 10.4. Latent Class Analysis (Students Positively Screened for at Least One BA)

#### 10.4.1. Latent Classes

Table S8 presents the results of the comparisons of LCA models in the sample of students with at least one BA (*n* = 327). The procedures for identifying the model best fitting to the data are described in Supplementary Materials (see also Figure S2).

**Figure 3:**
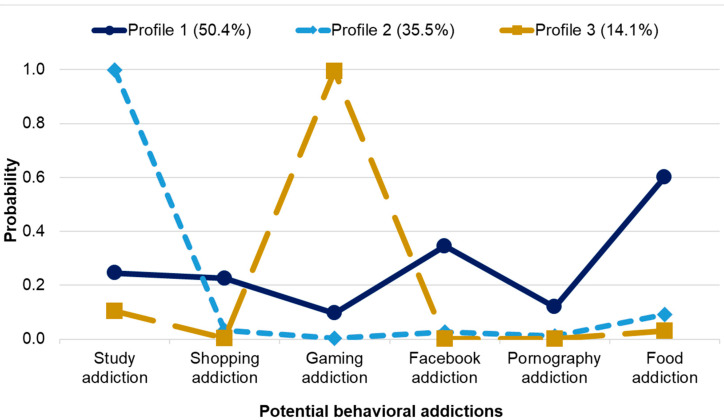
Three-Class LCA Model Results. *N* = 327.

The three-profile solution established by LCA is presented in Figure 3. Class 1 grouped persons with a heightened probability of almost all potential BAs (50.4%). Members of Class 2 had a very high probability of study addiction and a very low probability of other BAs (35.5%). The least numerous Class 3 (14.1%) comprised persons with a very high probability of gaming addiction and a very low probability of other BAs. Differences between classes in terms of levels of BAs are presented in Table S9.

#### 10.4.2. Latent Class Membership and External Variables

Table 8 presents the relationships between latent class membership and its potential predictors and consequences. The proportion of women to men was lower in students with a very high probability of gaming addiction (Class 3; 16.8% women), compared with members of other classes (Class 1; 60.8% and Class 2; 72.9%). Members of this profile were also less agreeable than members of the profile with a very high probability of study addiction (Class 2). Moreover, students in Class 2 were more conscientious than students in the class with a heightened probability of almost all BAs (Class 1) and students in the class with a very high probability of gaming addiction (Class 3; see Table 8).

For well-being indicators, the only significant differences between the classes were noted for hopelessness. Specifically, students who had a very high probability of study addiction (Class 2) scored lower on hopelessness than members of other classes.

### 10.5. LPA Classification Congruence between General Student Sample and Subsample

Since only LPA consistently replicated profiles, Table S10 shows classification congruence between the profiles in the general student sample and subsample. The majority of the cases in the subsample (74%) were previously classified in general proclivity and female-majority profiles in the general sample. General proclivity and female-majority profiles also had the highest congruence. While all cases from the male-majority profile from the general sample that entered the subsample were classified in the corresponding profile, 30% of the cases in the male-majority profile in the subsample were previously in the general proclivity profile in the general sample.

## 11. Discussion

Relatively strong theoretical assumptions and previous studies concerning co-occurring addictive behaviors suggested a cluster reflecting general proclivity to (BAs) and gender-specific clusters. LPA on the subsample mostly replicated the previous four profiles found in full sample: the first profile with elevated levels of study, Facebook, shopping, and food addictions; the second profile with elevated levels of gaming and pornography addictions; the third profile with the average levels of all addictions and low level of pornography addiction; and the fourth profile with the high levels of all addictions (H1 substantiated). The third profile in the subsample represents a cluster with relatively low levels compared with other profiles of particular BAs (see Table S3), and from this point of view constitutes a similar solution to the corresponding profile in the full sample. Most cases in this profile come from the corresponding profile in the full sample (see Table S10). Study addiction was the most prevalent addiction permeating and mostly dominating all profiles. The profiles identified in the subsample showed significantly fewer differences, especially in terms of the relationship with well-being, which is expected because they all reflect a more clinical subset of individuals with high risk for at least one BA. Still, the profile representing general proclivity for addictions scored significantly higher on hopelessness and anxiety and scored lower on health quality than most of the other profiles. A majority of the cases in the subsample were previously classified in general proclivity and female-majority profiles in the general sample, which suggests that these profiles represent and include the most clinically relevant cases. A majority of persons belonging to a given profile in the full sample were congruently classified into the corresponding profile in the subsample. Particularly accurate classifications were noted in female-majority and general proclivity profiles.

However, LCA on a general student sample did not replicate previous findings using LPA and showed only two classes: those having relatively high probabilities on all BAs and those with low probabilities on all BAs (H2 not substantiated). LCA on a subsample conflated profiles identified with LPA and classes found with LCA in the general student sample (H3 partially substantiated). Overall, LCA on dichotomized scores (screened positively vs. negatively) was less effective in identifying theoretically expected and clear groupings of BAs. It suggests that BAs can be investigated on the whole spectrum of behavior and that a person-centered approach might be more useful when it is based on continuous scores.

In the more clinical subsample, some of the patterns of potential antecedents and consequences were less pronounced, probably due to range restriction effects and decreased variability in the scores (H4 partially substantiated). Additionally, LCA generally produced fewer of the expected patterns of the potential risk factors and outcomes.

### 11.1. LPA vs. LCA

The comparison between LPA and LCA shows that dichotomizing continuous scores on addiction tests may limit the effectiveness of the analysis to identify clear and distinct clusters by limiting the available information. Dichotomizing continuous variables is a well-recognized practice to avoid in any statistical analyses [128,129,130]. A detailed overview of its negative consequences in medical research can be found elsewhere [131,132]. However, so far, little attention has been devoted to its consequences in the person-centered approach in the addiction field. The current study shows that well-defined and theoretically grounded profiles emerged in the general student sample when LPA was applied, which were largely replicated in a more clinical subsample and could not be identified with LCA. The latter method only distinguished two classes. The class with higher probabilities of all BAs included more women and the highest probability on food, study, and Facebook addictions. It suggests that LCA poorly differentiates the most severe group of general proclivity to addiction (predominantly male) and female-majority group, conflating these two distinct clusters found with LPA. Additionally, only emotional stability and narcissism differentiated these groups, showing that the predictive value of conscientiousness and extraversion was lost. In the subsample, LCA showed somewhat similar profiles to LPA but again conflating general proclivity to addiction (predominantly male) and female-majority group and showing highly “polarized” clusters with extreme cases of the high study addiction profile and the high gaming addiction profile. The study addiction class was more conscientious and less hopeless than the other two classes, and it was more agreeable than the high gaming addiction group. In conclusion, LCA showed inconsistent classes in comparison with LPA. The latter method showed highly consistent profiles in the general student sample and more clinical subsample, well-differentiated and grounded in theoretical assumptions.

The analysis of previous studies showed that most person-centered research on co-occurring addictive behaviors is based on LCA and produces mostly clusters differentiating general addiction severity or ad hoc identified clusters (see Table 2). These are very rarely or never replicated. They are also rarely grounded in strong theoretical assumptions and prior expectations. Sample types (e.g., general population vs. clinical) and sizes, as well as the choice of addictive behaviors analyzed, likely affect the results of these studies. However, the current study results suggest that this heterogeneity of results may be, to a considerable extent, associated with the limited information from dichotomous or dichotomized variables (e.g., addicted vs. non-addicted) on which LCA is based.

### 11.2. Prevalence and Co-Occurrence

Proper identification of theoretically expected clusters of BAs with the person-centered approach may be crucial for improving our understanding of the addictive process. In this context, a detailed analysis of prevalence and co-occurrence of the investigated BAs is particularly useful. Four major conclusions of high importance to the addictive disorders field can be drawn from the current study. First, the general proclivity to the addiction profile showing the worst well-being indicators in the subsample included 29.5% of individuals, meaning that it constituted about 8% of the total sample. Second, study addiction had the highest rates among all BAs, exceeding even food addiction, being up to about 4 to 7 times higher than some of the other BAs (shopping or pornography), constituting almost half of the cases in the subsample, and permeating all profiles. Third, individuals at risk for different BAs (Facebook, shopping, gaming, and pornography) also had a significant risk of food and study addictions. Fourth, individuals at risk of shopping and pornography addiction had a high risk for other BAs, including a high risk for such seemingly dissimilar behaviors as compulsive studying.

The most severe cases of BAs representing a general proclivity for addiction with clearly decreased well-being and the most pronounced personality risk factors constituted about 8% of the general student sample. It is almost 4 times less than the number of individuals who fulfilled the cutoff score for at least one addictive behavior (28.2% of the total sample). On the other hand, the LCA-based class representing a high risk for BAs in the general student sample also included about 9% of the total sample. However, only about half of the cases in this class were part of the subsample’s general proclivity to an addiction profile. The other half mostly included female-majority profile cases. First, this suggests that estimating prevalence rates of addictive disorders in a general population-based on screening for single BAs may significantly overestimate the rates of clinical cases of addiction. Moreover, these most severe cases constituted about 30% of the corresponding profile in the general student sample, meaning that most cases in this profile in the full sample are probably not clinically relevant. It shows that LPA on co-occurring BAs in the general population is probably not efficient enough to be used as a diagnostic method and might include a majority of false positives. This is also true for the gender-specific profiles. However, in conjunction with other criteria, such as fulfilling a cutoff score for at least one addictive behavior, it might prove useful in identifying the most severe cases of addiction. Second, the female-majority profile included some of the most severe cases identified in the general student sample by LCA, and generally, LCA conflated cases representing general proclivity and female-majority profiles. This suggests that the female-majority profile includes severe and probably clinically relevant cases of BAs. It was mostly not the case in relation to the male-majority profile, which almost exclusively included low severity cases in the general sample.

Study addiction, as well as work addiction, are consistently found to have the highest rates among all BAs [42,97,98,99]. Study addiction was the most or second-most prevalent addiction in all profiles, including male-majority. This result is counterintuitive but consistent with the way LPA operates. The identification of profiles uses information on the whole spectrum of behavior, and this way, a profile with high scores on gaming and pornography addiction can be distinguished. However, study addiction is the most prevalent addiction among those studied, and it is highly prevalent in all profiles. Because of that, the average score on study addiction in a clinical-like sample of those screened for at least one addictive behavior corresponds to a substantial number of study addiction cases based on cutoff score (almost half of the sample). Moreover, this is not a methodological fluke because the cutoff score, though not perfect, shows good validity. Those who are above the cutoff show significantly worse functioning, e.g., about 2 times higher depression and generalized anxiety rates [41]. It shows that if a person has some risk for addictive behavior (Facebook, shopping, gaming, pornography, food, etc.) in a population for which the main social role is studying, it is more likely that they will be addicted to studying too. Even if the behavior is ostensibly so different as gaming or pornography. Furthermore, study and work addiction have been linked to anankastia/ obsessive-compulsive personality disorder (OCPD), which is also the most prevalent personality disorder in the general population (see [71]). Moreover, study addiction was consistently negatively associated with all indicators of well-being in a general sample and in the previous research [41,42,67]. Additionally, the estimates of Facebook, shopping, food, and gaming addictions are largely consistent with other studies based on similar methodology [45,79,87,92,106].

The highest co-occurrences were noted among food, shopping, and Facebook addictions, as well as between gaming and pornography addictions. Moreover, study addiction and food addiction were highly prevalent among individuals with all other addictions (on average, in about 30% of cases; see Table 5), which, to some extent, is probably related to their high prevalence in general. It is an especially interesting result because it seems to suggest that among individuals with compulsive tendencies, there is a high risk for two particular addictive behaviors: i) one related to fundamental physiological need (food), and ii) one related to basic social role (study) (see clusters found in [28]). Both behaviors have essential meaning to survival in modern society—biological and social. Additionally, both problematic behaviors pose the same crucial question, which mostly does not pertain to other addictions (or has considerably less importance): how to regulate behavior that cannot be stopped completely?

On the other hand, pornography and shopping addictions were behaviors with the highest co-occurrence (on average of more than 25%; see Table 5) among all other addictions, including study addiction. These behaviors are commonly identified as products of modern society, enabling highly indulgent hedonistic activity, and are related to impulsivity [36,133,134,135]. It seems to suggest that individuals frequently pandering to these behaviors may be at higher risk for other addictive behaviors. This result also seems to support further the notion that impulsivity and compulsivity are significantly associated with addictive behaviors [16,136]. Probably more attention should be devoted to discerning impulsive and compulsive urges in order not to misclassify behaviors as pathological, especially in the cases in which normal impulsive behavior does not cause evident harm (e.g., sporadic impulsive buying behavior or pornography consumption).

Study addiction is the second-most prevalent addiction in the profile with a general proclivity to addictions (see Table S4); however, most of its cases are classified in the female-majority profile (see Table S4). This suggests that study addiction is both typical for individuals with a proclivity to addictive behaviors (mostly men) and a highly specific type of addiction (predominant among women). Probably at least two major profiles of study addicts may be identified and are related to gender differences [1].

### 11.3. Implications for Research and Interventions

A person-centered approach is a method of investigating addictive behaviors complementary to the latent trait model. LCA on dichotomized scores (screened positively vs. negatively) was less effective in identifying clear patterns of interrelationships between BAs than LPA. This study suggests that profiles identified within the full spectrum of behavior with LPA are similar to profiles found in the potentially more clinical subsample of BAs. It is consistent with the assumption that addictions can be studied along the continuum of severity of the behavior. Based on these results, it can be recommended to investigate profiles of BAs and their co-occurrences with continuous variables in samples representing the general population. It has an important advantage, as it could overcome limitations related to small sample sizes typical for clinical samples. Furthermore, continuous scores on valid and reliable addiction scales provide more information than dichotomized scores or clinical classifications, and they limit the problems of (i) borderline cases (slightly above and slightly below the cutoff score), (ii) misdiagnoses (false positives and false negatives), (iii) false homogeneity among those classified as addicted in terms of severity of symptoms, and (iv) false homogeneity among those classified as non-addicted in terms of risk levels. It has to be emphasized, however, that the validity of such analyses is dependent to a large degree on the validity of the assessment tools. In recent years, there have been substantial developments in the measurement of psychopathological phenomena, including addiction and BAs. Still, there is room for considerable improvement [137,138,139,140].

This and previous studies have shown that there are different clusters of individuals manifesting specific risks for addictions. Most notably, a cluster representing general proclivity for addiction with high co-occurring risks of numerous addictive behaviors requires the most attention from addiction professionals and researchers. Moreover, clusters representing gender-specific addictions need more research to establish whether these are individuals on a trajectory to full-blown addiction syndrome or cases of people highly engaged in a non-pathological way in particular sets of behaviors. It is especially important in relation to gaming and pornography consumption, as these are typically recognized as major threats despite quite a lot of evidence that, in most cases, there is limited empirical support suggesting their negative consequences on a population level [52,141]. On the other hand, such socially valued behaviors as studying show unambiguous relations to depression, anxiety, and other psychosocial harms when they become compulsive [41,67]. Moreover, some data indicate that they may progress with time [96] and are longitudinally related to work addiction after graduation and entering the labor market [142]. This behavior is evidently more common among women in a profile including shopping, social networking, and food.

A general proclivity profile includes behaviors present in female- and male-majority profiles. This suggests that within a particular behavior, different clusters of risk or addiction levels can be distinguished. It was repeatedly shown for shopping addiction, food addiction [143], social networking sites addiction [144], gaming addiction [17], and pornography.

The female-majority profile is probably least recognized as a genuine addiction profile, and none of the prevalent behaviors constituting it are currently officially recognized in the formal classification systems as addiction [145]. It may be related to the fact that the behaviors comprising it may not be typically recognized by society and researchers as addictive behaviors. Partly, their harmful potential may be overlooked due to low sensation-seeking or impulsivity associated with the behaviors. Additionally, individuals engaging excessively in these behaviors may not immediately suffer pronounced impairments, which makes it more difficult to recognize their negative consequences. Furthermore, higher conscientiousness may be a protective factor against immediate health issues, and higher extraversion may protect against social exclusion and loneliness. This protective personality effect, however, may mask the negative consequences of addiction, resulting in significantly non-optimal functioning but still without immediately visible harm. Though, it needs to be taken into account that when controlling for personality and demographics in the analyses, the relationship of this profile with well-being did not change significantly [1]. Additionally, it seems very likely that harm in the case of this profile may take a longer time to become visible, which paradoxically may be related to worse outcomes because with time the addictive pattern becomes rigid and resistant to change. Such could be the case of work addiction, in which harm may be first most pronounced in relation to family functioning [63,146] or coworkers and the recipients of work [65,147]. Likewise, shopping addiction or Facebook addiction may result in family dysfunction, including child neglect, which is difficult to measure in self-report studies.

### 11.4. Strengths and Limitations

To the Authors’ knowledge, the current study is the first to investigate the latent profiles and latent classes based on highly gender-differentiated BAs. A range of their potential antecedents and consequences were analyzed. A relatively large sample size provided high statistical power. Commonly used, valid, and reliable psychometric tools were included. LPA has identified and replicated three types of potential BAs severity profiles, which have considerable potential for generality. Moreover, this study has provided important information on the differences between results obtained with LPA and LCA, suggesting the higher effectiveness of LPA in identifying clear patterns of clusters in the same samples. Therefore, the study significantly adds to the addiction literature, particularly in the area of research related to BAs. The results increase our understanding of the nature of the addictive process.

The study also has some limitations. All data were self-reported, resulting in a susceptibility to the usual weaknesses of such data (e.g., common method, social desirability, and recall biases). Although the used cutoff scores are well-grounded in the modern nomological systems, are commonly used, and were validated psychometrically, they are all arbitrary. The subsample was based on psychometric cutoff scores and not clinically derived. Therefore, it is likely that a considerable number of cases do not fulfill the criteria for a disorder because screening tools, in general, overestimate the number of addiction cases [12], and particularly, the gaming addiction scale that was used is known to overestimate the prevalence of internet gaming disorder [78]. Moreover, while study addiction has been conceptualized as a potential early form of work addiction [41,67] and was shown to be related to it in a longitudinal study [96], there are evident differences in the risk factors of these behaviors. For example, there are 2 to 3 times more female students addicted to studying, in comparison with male students, and no such gender differences are observed in work addiction; additionally, there is a positive relationship between attention deficit hyperactivity disorder (ADHD) and work addiction and no such clear association between ADHD and study addiction [1,41,42]. The study did not include some of the other highly prevalent potential BAs, such as exercise or love addiction. Moreover, while opinions still diverge, food addiction is gradually being recognized as a substance addiction rather than behavioral eating addiction [148,149,150,151]. The sample size for multinomial logistic regression analysis of potential profile predictors in a subsample could be considered borderline acceptable according to some recent recommendations [152]. At the same time, it points to the benefits of conducting LPA on general population samples, as it may improve the performance of such analyses due to a larger sample size in comparison with clinical samples. Finally, due to the cross-sectional design, no causal inferences can be made. Commonly, personality traits and sociodemographics are investigated as antecedents of potential BAs, and well-being indicators are typically considered the consequences of potential BAs. Still, it should be kept in mind that variables such as personality or well-being can have bidirectional relationships with potential BAs.

## 12. Conclusions and Future Research Directions

LPA on a sample of students positively screened for at least one BA mostly replicated previous findings in a general student sample. They suggest a profile reflecting general proclivity to BAs and gender-specific profiles of BAs. According to the results, men show a higher proclivity towards gaming and pornography and women towards study, Facebook, shopping, and food addictions. It suggests that profiles identified within the full spectrum of behavior are similar to profiles found in a potentially more clinical subsample of BAs. Our findings are consistent with the assumption that addictions can be studied along the continuum of severity of the behavior. The profiles identified in the subsample showed significantly fewer differences, especially in terms of the relationship with well-being, which is expected because they all reflect a more clinical subset of individuals with high risk for at least one BA and a limited variability of scores in comparison with the general student sample. Still, the profile representing general proclivity for BAs scored significantly higher on hopelessness and anxiety and lower on health quality than most of the other profiles.

Moreover, LCA on a full sample did not replicate previous findings using LPA. LCA on dichotomized scores (screened positively vs. negatively) was less effective in identifying clear and replicable groupings based on relationships among various BAs. Person-centered analyses might be more useful when they are based on continuous scores. Measuring scores on the whole spectrum of addictive behavior is recommended, and reducing continuous scores into few categories, especially only two (addicted vs. non-addicted), is not advisable.

Finally, a detailed analysis of the prevalence and co-occurrence of the investigated BAs suggests three major conclusions of high importance to the addictive disorders field. First, studies investigating multiple addictive behaviors may overestimate their total prevalence in the population. Second, study addiction has the highest rates among all BAs, exceeding even food addiction, permeating all profiles, and is most or second-most prevalent in each of them. Third, individuals at risk for different BAs (Facebook, shopping, gaming, and pornography) have a significant risk of food and study addictions. Fourth, individuals at risk of shopping and pornography addictions have a high risk for other addictive behaviors, including a high risk for such seemingly dissimilar behaviors as studying. Based on these findings, identifying individuals with high risk for multiple addictive behaviors is recommended to improve prevention programs and to understand the addictive process. Specifically, diagnosis of one addictive behavior should be complemented by detailed inquiry into other potential addictions. Consequently, the treatment process should address general proclivity to addiction to assure that one addictive behavior will not be substituted with another, including those such seemingly less damaging as study addiction. As the most prevalent and almost completely unrecognized potential BA, study addiction urgently needs widespread acknowledgment, more research, and potentially population-based prevention programs and proper policy responses.

Future studies should investigate a wider range of addictive behaviors with LPA and further compare results based on LPA and LCA. Additionally, a more theoretically derived investigation of profiles of addictive behaviors based on a confirmatory approach and enabling more generalizations and replications based on person-centered analyses is warranted. Currently, the overwhelming majority of studies on co-occurring addictive behaviors are based on LCA and produce either clusters differentiating general addiction risk or ad hoc established clusters that are never or very rarely replicated. This, to a considerable extent, may be due to limited information associated with dichotomous variables (e.g., addicted vs. not addicted) used in LCA.

## Figures and Tables

**Table 1:** Description of the Four Profiles of Behavioral Addictions (BAs).

	Profiles			
Levels of BAs	Elevated levels of study, Facebook, shopping, and food addictions	Elevated levels of gaming and pornography addictions	Elevated levels of all BAs	Average or low levels of all BAs
Prevalence	28.6%	24.6%	23.1%	23.7%
Label	Female-majority	Male-majority	General proclivity towards BAs	Low risk of BAs
Potential predictors and consequences				
Gender (women)	92%	18%	34%	58%
Emotional stability			Lowest	
Narcissism			Highest	
Conscientiousness	Highest	Low	Low	
Extraversion	Highest			
Quality of life			Lowest	
Health quality		Highest	Lowest	
Sleep quality			Lowest	
Perceived stress			Highest	
Anxiety		Low	Highest	
Hopelessness			Highest	

Note. The table is an elaboration of the results presented in [1]. The values of the potential predictors and consequences are only reported if they showed statistically significant differences among profiles (*p* < 0.05). Some of the values could still be relatively high or low; however, they were not statistically different among profiles in this sample.

**Table 2:** Studies Using Person-Centered Analyses of Various Addictive Behaviors (Clusters Identified Based on at Least Two Different Behaviors).

Type of Addiction	Sample Type	Sample Size	Analysis	Measurement	Clusters Represent	Reference
study, shopping, gaming, Facebook, pornography, food	Sample of general undergraduate student population	1182	LPA	Continuous scores on addiction scales	Addiction severity Gender differences	Charzyńska et al., 2021 [1]
internet gaming, social media (impulsiveness and psychopathology)	General adolescent population sample	643	LPA	Continuous scores on addiction scales	Addiction severity/risk Age differences	Cerniglia et al., 2019 [17]
alcohol, tobacco, cannabis, gambling	General adolescent population sample	1644	LCA	Frequency of behavior and dichotomized continuous addiction scales	Addiction risk Gender differences Complex patterns	Martínez-Loredo et al., 2019 [16]
alcohol, drugs, smoking, gambling	General student population sample	2139	LCA	Frequency of behavior and continuous addiction scales recoded as categorical	Probability of behavior/addiction risk	Kairouz et al., 2018 [25]
gambling, sexual addiction, buying, videogame use, eating disorders	Clinical sample	302	Growth Mixture Models and LCA	Addiction Severity Index	Severity of addiction Level of behavior Complex patterns	Montourcy et al., 2018 [26]
cigarettes, alcohol, hard drugs, eating, gambling, Internet, love, sex, exercise, work, shopping	General adolescent population sample	715 Russian 811 Spanish	LCA	Dichotomous responses on addiction questions	Addiction presence	Tsai et al., 2017 [27]
alcohol, tobacco, marijuana, cocaine, gambling, eating, shopping, sex, video gaming, work	General population sample	2728	Hierarchical cluster analyses	Occurrence of excessive behavior: dichotomized	Probability of behavior/addiction risk Gender differences Complex patterns	Konkolý Thege et al., 2016 [28]
alcohol, tobacco, cannabis, other drugs, gambling, shopping, exercise, Internet, mobile phone, work, overeating	General population sample	770	LCA	Frequency of excessive behavior dichotomized	Probability of behavior/addiction risk	Deleuze et al., 2015 [15]
cigarettes, alcohol, hard drugs, shopping, gambling, Internet, love, sex, eating, work, exercise	Sample of former alternative high school youth at risk for addictions	538	LCA, LTA (latent transition analysis)	Dichotomous responses on addiction questions	Addiction presence Probability of transitioning from one class to another	Sussman et al., 2015 [29]
cigarettes, alcohol, other/hard drugs, eating, gambling, Internet, shopping, love, sex, exercise, work	Sample of former alternative high school youth at risk for addictions	717	LCA	Dichotomous responses on addiction questions	Addiction presence	Sussman et al., 2014 [30]
Internet, smartphone	General university student population sample	448	LCA	Continuous scores on addiction scales recoded into categorical (no description of how)	Level of behavior/addiction risk	Mok et al., 2014 [31]
alcohol, drugs, tobacco, cannabis, substitute opiate prescribing, behavioral addiction without eating disorders	Clinical sample	301	Cluster analysis	Addiction severity coded as categorical	Severity of addiction/addiction risk Complex patterns	Combes 2014 [32]

Note. Some studies were not included because there was no English version of the paper or no access to an accurate description of the methodology (Pikó & Kiss 2019 [33]; Quinn et al., 2019 [34]).

**Table 3:** Characteristics of the Study Measures.

Variable	Measure	Number of Items	Range of Response Options	Reference
*Behavioral addictions*				
Study addiction	Bergen Study Addiction Scale (BStAS)	7	*never* (1) to *always* (5)	Atroszko et al., 2015 [67]
Shopping addiction	Bergen Shopping Addiction Scale (BSAS)	7	*completely disagree* (1) to *completely agree* (5)	Andreassen et al., 2015 [60];
Gaming addiction	Game Addiction Scale (GAS)	7	*never* (1) to *very often* (5)	Lemmens, Valkenburg, and Peter 2009 [106];
Facebook addiction	Bergen Facebook Addiction Scale (BFAS)	6	*very rarely* (1) to *very often* (5)	Andreassen, Torsheim et al., 2012 [107];
Pornography addiction	Compulsive Pornography Consumption (CPC) Scale	6	*never* (1) to *very frequently* (5)	Noor, Rosser, and Erickson 2014 [108]
Food addiction	Modified Yale Food Addiction Scale (mYFAS)	9	*never* (1) to *4 or more times a week or daily* (5)	Lemeshow et al., 2016 [109]
*Personality*				
Big Five personality	Ten Item Personality Inventory (TIPI)	10	*strongly disagree* (1) to *strongly agree* (7)	Gosling, Rentfrow, and Swann 2003 [110]
Narcissism	Single-Item Narcissism Scale (SINS)	1	*no* (1) to *yes* (9)	Konrath, Meier, and Bushman 2014 [111]
*Functioning*				
General quality of life, health quality, and sleep quality	Items based on the WHOQOL-BREF	3	*very dissatisfied* (1) to *very satisfied* (9) or *very poor* (1) to *very good* (9)	Atroszko 2015 [41] Skevington et al., 2004 [112]
Perceived stress	Perceived Stress Scale (PSS-4)	4	*never* (1) to *very often* (5)	Cohen, Kamarck, and Mermelstein 1983 [113]
Short anxiety scale	Short Anxiety Scale (SAS)	5	*never (1) to most of the time (4).*	Clarke et al., 2008 [114]
Hopelessness	Short Hopelessness Scale (SHS)	4	*I totally disagree* (1) to *I totally agree* (6)	Clarke et al., 2008 [114]

Note: Detailed information on the Polish adaptations and validity and reliability of all measures can be found in [1].

**Table 4:** Mean, Standard Deviations, and Correlations Between the Study Variables.

Variables	M	SD	Range	(1)	(2)	(3)	(4)	(5)	(6)	(7)	(8)	(9)	(10)	(11)	(12)
(1) Study addiction ^a^	21.11	6.26	7–35	1											
(2) Shopping addiction ^a^	14.83	6.53	7–35	−0.01	1										
(3) Gaming addiction ^a^	13.93	8.12	7–35	−0.26 ***	0.10	1									
(4) Facebook addiction ^a^	14.85	6.40	6–30	0.04	0.31 ***	−0.06	1								
(5) Pornography addiction ^a^	9.95	5.75	6–30	−0.11	0.25 ***	0.44 ***	0.15 **	1							
(6) Food addiction ^a^	22.67	8.56	9–45	−0.03	0.28 ***	0.05	0.34 ***	0.14 *	1						
(7) Study addiction ^b^	49.2% ^c^	–	–	0.79 ***	−0.17 **	−0.32 ***	−0.15 **	−0.20 ***	−0.24 ***	1					
(8) Shopping addiction ^b^	12.5% ^c^	–	–	−0.10	.67 ***	0.07	0.11 *	0.14 *	0.12 *	−0.15 **	1				
(9) Gaming addiction ^b^	19.0% ^c^	–	–	−0.28 ***	0.00	0.80 ***	−0.13 *	0.21 ***	−0.04	−0.29 ***	0.03	1			
(10) Facebook addiction ^b^	18.3% ^c^	–	–	−0.05	0.08	−0.08	0.71 ***	0.03	0.17 **	−0.17 **	0.04	−0.09	1		
(11) Pornography addiction ^b^	6.4% ^c^	–	–	−0.02	0.14 *	0.11	0.10	0.65 ***	0.06	−0.08	0.09	0.00	0.07	1	
(12) Food addiction ^b^	33.9% ^c^	–	–	−0.08	0.16 **	−0.02	0.26 ***	0.06	0.79 ***	−0.23 ***	0.06	−0.12 *	0.08	0.00	1
(13) Gender	58.1% ^d^	–	–	−0.15 **	0.09	0.56 ***	−0.07	0.61 ***	−0.04	−0.20 ***	0.05	0.36 ***	−0.05	0.27 ***	−0.06
(14) Age	20.55	1.66	18–30	0.06	0.12 *	0.01	0.09	0.00	0.13 *	−0.01	.04	0.04	0.01	−0.08	0.13 *
(15) Extraversion	8.63	2.99	2–14	0.10	0.02	−0.14 *	0.10	−0.15 **	0.00	0.12 *	−0.01	−0.11	0.01	−0.04	−0.01
(16) Agreeableness	9.31	2.46	2–14	0.08	−0.24 ***	−0.07	.00	−0.06	−0.13 *	0.10	−0.17 **	−0.09	0.07	−0.10	−0.09
(17) Conscientiousness	9.18	2.89	2–14	0.33 ***	−0.13 *	−0.28 ***	−0.05	−0.18 **	−0.16 **	0.39 ***	−0.10	−0.21 ***	−0.03	−0.04	−0.07
(18) Emotional stability	7.71	2.77	2–14	0.01	−0.03	0.12 *	−0.03	0.08	−0.12 *	−0.02	−0.01	0.07	−0.04	0.02	−0.09
(19) Openness	9.80	2.29	2–14	−0.04	−0.05	−0.08	−0.06	−0.21 ***	−0.09	.04	−0.02	0.01	0.02	−0.17 **	−0.06
(20) Narcissism	4.24	2.38	1–9	−0.03	0.19 ***	0.06	0.15 **	0.17 **	0.11	−0.10	0.14 *	0.03	0.05	0.18 **	0.06
(21) General quality of life	6.70	1.48	1–9	0.05	0.01	−0.04	0.00	−0.01	−0.12	0.08	0.03	−0.06	−0.03	−0.01	−0.07
(22) Health quality	5.59	2.20	1–9	−0.10	−0.10	−0.03	−0.09	−0.09	−0.18 **	−0.06	−0.01	0.02	−0.07	−0.13 *	0.04
(23) Sleep quality	4.60	2.20	1–9	−12 *	0.04	−0.05	0.05	0.01	−0.08	−0.15 **	0.01	−0.05	0.06	−0.02	−0.05
(24) Perceived stress	12.11	2.95	4–20	0.01	−0.05	0.02	0.10	0.03	0.16 **	−0.02	−0.03	0.06	0.10	0.02	0.13 *
(25) Anxiety	10.90	3.06	5–20	0.09	0.11 *	0.07	0.16 **	0.03	0.23 ***	−0.01	0.01	0.01	0.12 *	0.01	0.16 **
(26) Hopelessness	10.35	4.73	4–24	−0.07	0.16 **	0.17 **	0.15 **	0.17 **	0.25 ***	−0.16 **	0.07	0.14 *	0.12 *	0.08	0.20 ***

Note. * *p*  < 0.05; ** *p*  < 0.01; *** *p*  <  0.001. ^a^ Continuous scores. ^b^ Dichotomous scores. ^c^ For BAs treated dichotomously, the values present the percentage of positively screened participants. ^d^ For gender, the values present the percentage of women. Gender was dummy coded (0 = women, 1 = men). BAs from (1) to (6) were treated as continuous variables; BAs from (7) to (12) were dummy-coded (0 = no; 1 = yes). Pearson’s correlation coefficient was used to calculate the relationship between continuous variables and between continuous and dichotomous variables; for two dichotomous variables, the Phi correlation coefficient was used. M = mean; SD = standard deviation. N = 327.

**Table 5:** Co-Occurrence of Potential BAs.

Potential BAs	Percentage of co-Occurrence of a Given BA						Average Co-Occurrence of Other BAs
	Study Addiction	Shopping Addiction	Gaming Addiction	Facebook Addiction	Pornography Addiction	Food Addiction	
Study addiction	–	7.5%	7.5%	11.8%	4.4%	23.0%	10.8%
Shopping addiction	29.3%	–	22.0%	22.0%	12.2%	41.5%	25.4%
Gaming addiction	19.4%	14.5%	–	11.3%	6.5%	22.6%	14.8%
Facebook addiction	31.7%	15.0%	11.7%	–	10.0%	41.7%	22.0%
Pornography addiction	33.3%	23.8%	19.1%	28.6%	–	33.3%	27.6%
Food addiction	33.3%	15.3%	12.6%	22.5%	6.3%	–	18.0%
Average co-occurrence of a given BA	29.4%	15.2%	14.6%	19.2%	7.9%	32.4%	19.8%

Note. N = 327.

**Table 6:** Potential Predictors and Outcomes of LPA Membership: Comparison of Profiles.

Sociodemographics and Personality	Overall Wald Test	Standardized Scores				Wald’s Values for the Pairwise Comparisons among Profiles					
		z_1_	z_2_	z_3_	z_4_	1 vs. 2	1 vs. 3	1 vs. 4	2 vs. 3	2 vs. 4	3 vs. 4
Gender ^a^	76.93 ***	96.33%	22.90%	77.72%	29.62%	**52.75**	**8.80**	**34.21**	**33.09**	**6.10**	**14.77**
Age	10.80 **	0.11	−0.33	−0.14	0.18	**8.22**	3.34	2.08	3.18	**3.93**	0.08
Extraversion	1.58	0.16	−0.15	0.11	−0.15	1.37	0.77	0.34	0.27	0.45	0.05
Agreeableness	0.51	0.08	0.06	−0.04	−0.12	0.24	0.01	0.32	0.27	0.00	0.35
Conscientiousness	13.64 **	0.39	−0.04	−0.16	−0.31	1.50	**7.82**	**11.92**	0.27	1.27	0.87
Emotional stability	2.15	0.02	0.00	−0.18	0.09	1.69	0.63	0.44	0.51	0.87	0.01
Openness	6.06	0.08	0.04	0.30	−0.29	1.21	2.89	0.32	0.06	2.67	**3.94**
Narcissism	5.92	−0.13	−0.21	−0.06	0.33	2.85	0.10	0.86	0.06	**5.69**	**3.93**
Well-being indicators											
General quality of life	1.47	0.03	0.00	0.11	−0.10	0.03	0.25	0.77	0.33	0.30	1.33
Health quality	12.97 **	0.10	0.30	−0.07	−0.29	1.66	0.98	**7.47**	3.47	**11.44**	1.45
Sleep quality	0.13	0.01	−0.03	−0.03	0.02	0.05	0.06	0.00	0.00	0.06	0.07
Perceived stress	2.84	−0.08	−0.01	−0.06	0.13	0.22	0.02	2.55	0.06	0.70	1.02
Anxiety	11.44 **	−0.11	−0.27	0.16	0.22	0.97	1.97	**5.44**	**4.30**	**9.28**	0.10
Hopelessness	10.51 *	−0.11	−0.17	−0.09	0.29	0.17	0.02	**7.90**	0.18	**7.57**	3.77

Note. * *p*  < 0.05; ** *p*  <  0.01; *** *p*  <  0.001. ^a^ For gender, the percentage of women in a given profile is presented. All continuous variables are presented in a standardized form. A dummy variable was created for gender (0 = women; 1 = men). For profile comparisons, a value of the Wald statistic higher than 3.84/6.63/10.83 indicates a significance level of 0.05/0.01/0.001, respectively. Significant differences between the profiles are in bold. Profile 1: elevated levels of study, Facebook, shopping, and food addictions (33.7%); Profile 2: elevated levels of gaming and pornography addictions (20.1%); Profile 3: average or lower than average levels of all BAs (16.7%); Profile 4: elevated levels of most potential BAs (29.5%). N = 327.

**Table 7:** Potential Predictors and Outcomes of LPA Membership: Comparison of Classes.

Sociodemographics and Personality	Wald Test	Standardized Score	
		z_1_	z_2_
Gender ^a^	5.22 *	51.38%	62.23%
Age	5.65 *	−0.03	0.36
Extraversion	0.59	0.02	−0.20
Agreeableness	1.54	0.06	−0.56
Conscientiousness	1.45	0.02	−0.20
Emotional stability	9.76 **	0.06	−0.58
Openness	2.40	0.03	−0.25
Narcissism	10.74 **	−0.06	0.58
Well-being indicators			
General quality of life	9.40 **	0.05	−0.48
Health quality	25.52 ***	0.07	−0.71
Sleep quality	26.60 ***	0.07	−0.66
Perceived stress	63.87 ***	−0.10	1.06
Anxiety	48.03 ***	−0.10	0.99
Hopelessness	46.28 ***	−0.10	1.02

Note. * *p*  < 0.05; ** *p*  <  0.01; *** *p*  <  0.001. ^a^ For gender, the values present the percentage of women in a given class. Class 1: a low probability of potential BAs (91.0%); Class 2: the heightened probability of all BAs (9.0%). N = 1157.

**Table 8:** Potential Predictors and Outcomes of LCA Membership: Comparison of Classes.

Sociodemographics and Personality	Overall Wald Test	Standardized Scores			Wald’s Values for the Pairwise Comparisons among Classes		
		z_1_	z_2_	z_3_	1 vs. 2	1 vs. 3	2 vs. 3
Gender ^a^	20.99 ***	60.84%	72.89%	16.75%	0.23	**19.51**	**17.30**
Age	1.83	0.06	−0.06	−0.03	0.43	1.68	0.47
Extraversion	2.35	−0.02	0.15	−0.34	0.35	1.55	2.32
Agreeableness	6.47 *	−0.13	0.25	−0.19	3.02	1.74	**6.05**
Conscientiousness	36.07 ***	−0.17	0.50	−0.64	**24.58**	**5.99**	**29.21**
Emotional stability	1.38	−0.06	0.04	0.14	0.31	0.89	1.36
Openness	3.15	−0.06	0.11	−0.05	0.19	3.15	1.38
Narcissism	5.83	0.16	−0.24	0.03	3.82	3.06	0.03
Well-being indicators							
General quality of life	1.51	−0.02	0.08	−0.13	0.57	0.38	1.40
Health quality	1.01	−0.05	0.03	0.11	0.44	0.91	0.20
Sleep quality	2.41	0.09	−0.12	−0.01	2.41	0.24	0.41
Perceived stress	2.07	0.09	−0.11	−0.03	2.01	0.52	0.20
Anxiety	3.67	0.11	−0.10	−0.15	2.32	2.69	0.08
Hopelessness	16.24 ***	0.18	−0.32	0.16	**14.24**	0.03	**8.65**

Note. * *p*  <  0.05; *** *p*  <  0.001. ^a^ For gender, the percentage of women in a given class is presented. All continuous variables are presented in a standardized form. A dummy variable was created for gender (0 = women; 1 = men). For class comparisons, a value of the Wald statistic higher than 3.84/6.63/10.83 indicates a significance level of 0.05/0.01/0.001, respectively. Significant differences between the classes are in bold. Class 1: the heightened probability of almost all potential BAs (50.4%); Class 2: a very high probability of study addiction and a very low probability of other BAs (35.5%); Class 3: a very high probability of gaming addiction and a very low probability of other BAs (14.1%). N = 327.

## Data Availability

The data presented in this study are available on request from the corresponding author. The data are not publicly available due to the fact that participants were not informed while obtaining informed consent that the data will be made publicly available.

## Supplementary Materials

The following Supplementary Materials are available online at https://www.mdpi.com/article/10.3390/ijerph18168590/s1, Table S1: Comparisons Between Persons Without BAs and Persons with at Least One BA, Table S2: Comparison of LPA Models for Persons with at Least One Behavioral Addiction, Table S3: Profile Comparisons for the Four-Profile Solution, Table S4: Prevalence and Co-Occurrence of Potential Behavioral Addictions (BAs) among Four Latent Profiles, Table S5: The Frequency of the Number of BAs among the Latent Profiles, Table S6: Summary Comparison of LCA Models for the Full Sample, Table S7: Class Comparisons for the Two-Class Solution, Table S8: Comparison of LCA Models for Persons with at Least One Behavioral Addiction, Table S9: Class Comparisons for the Three-Class Solution, Table S10: The Accuracy of Classification of Cases in General Student Sample and Subsample Positively Screened for BAs Based on LPA, Figure S1: Elbow Plot of the BIC, CAIC, and SABIC Values for the LPA Solutions, Figure S2: Elbow Plot of the BIC, CAIC, and SABIC Values for the LCA Solutions.
