# Supplementary material for: Subpopulations of Addictive Behaviors in Different Sample Types and Their Relationships with Gender, Personality, and Well-Being: Latent Profile vs. Latent Class Analysis
Source: Int J Environ Res Public Health. 2021 Aug 14;18(16):8590. doi: 10.3390/ijerph18168590 (PMC8394473; doi:10.3390/ijerph18168590)
Supplement: Supplementary file 1 [file ijerph-18-08590-s001.zip › ijerph-1246920-supplementary.pdf]

Supplementary Materials

Prevalence of Behavioral Addictions (BAs): Extended Description

Review papers show that the prevalence of Internet gaming disorder (IGD) ranges from 0.7% to 15.6% [79], 0.7% to 27.5% [80], and even 0.21% to 57.50% in general populations [81]. Most studies are conducted in the Republic of Korea, China, and the USA. The pooled prevalence of IGD among adolescents in 16 studies was 4.6% [47], similar to 4.7% in the general population [79]. However, data from nationally representative samples suggest that the prevalence is considerably lower in the general population (only 0.7% to 1.4% of the gamers population [82,83], which in turn constitutes only a portion of the general population).

The prevalence estimates of social networking sites (SNS) addiction range from 5% to 25% depending on the used cutoff score, and depend on whether it is measured in collectivist or individualist nations [84]. The surveys on representative samples showed that 4.5% of persons belonged to the at-risk group for problematic social media use in an adolescents' sample in Hungary [85], and 2.9% compulsively used social networking sites in an adult sample in Belgium [86]. The prevalence of social media addiction is typically higher than Facebook addiction as measured in the same sample, because it includes other social media such as Instagram or Snapchat, and also comprises multi-site addiction [87]. For example, the prevalence of Facebook addiction ranged from 1.6% among Nigerian University undergraduates [88] to 41.8% among Thai adolescents (high school students; [89]). It should be noted

that such a low estimate in the Nigerian sample may be the result of a low level of Internet access in the respondents' country [88]. Also, Internet-related addictive behaviors tend to be more prevalent in Asian countries [84].

The estimates of the prevalence of compulsive buying in representative studies range from 1% to 8.1%, with a mean pooled estimate of 4.9% [12]. The estimates are considerably higher among university student samples (8.3%), in adult non-representative samples (12.3%), and in shopping-specific samples (16.2%)[12]. Food addiction prevalence estimates range from 2.6% among Dutch adolescents [90] to 56.8% among obese patients with binge eating disorder [91]. Meta-analysis showed that the weighted mean prevalence based on Yale Food Addiction Scale was 19.9% [46]. However, the analyzed studies included samples of predominantly female, overweight/obese, and adults aged over 35 years; therefore, not representative for a general population. In a nationally representative sample in Germany, the prevalence was 7.9% [92]. Researchers seem to be most reluctant to provide problematic pornography estimates [93,94], probably due to the highly controversial nature of this behavior and its investigation [95].

Finally, study addiction is conceptualized as a potential early form of work addiction [41,67], and was shown to be related to it in a longitudinal study [96]. As such, it is the least investigated potential addictive behavior among those included in this study. However, study addiction, as well as work addiction, are consistently found to have similar prevalence rates and to be highest among all BAs (about 10% in studying and working populations respectively; [42,97-99]) comparable maybe

only with food addiction [46,92]. Furthermore, study and work addition have been linked to anankastia/ obsessive-compulsive personality disorder (OCPD), which is also the most prevalent personality disorder in the general population (see [71]). A nationally representative survey among employees in Norway showed an 8.3% prevalence of work addiction [100], which was very similar to the prevalence of study addiction (9.7%) in a large sample (n = 2558) from various universities and faculties in Norway [41]. In Poland, rates varied from 6.4% in convenience samples collected during classes at university to 14.2% in samples collected online [41], and prevalence was 15.4% among high school students [42]. In a specific sample of music academies' students, the prevalence was very similar (16.0% [97]). These are again very similar to the estimates of the prevalence of work addiction in Poland (17.4%) based on an analogous measure using common components of addiction [101]. In India, study addiction was found among 17.1% of high school students [102].

Latent Profiles (Students Positively Screened for at Least One BA)

The values of model fit statistics decreased with the addition of the profiles. Thus, we prepared the elbow plot to detect the point at which the decrease in the fits became negligible [148]. For each information criterion, the plot flattened out after the fourth profile, suggesting that the four-profile solution fit the data best (Figure S1). Therefore, we further examined this profile and adjacent three- and five-profile solutions. Adding a fourth profile resulted in identifying four well-defined qualitatively distinct profiles, which confirmed the added value of the four-profile solution compared to the three-profile solution. By contrast, a closer look at the five-

profile solution showed that adding the fifth profile brought no new information to the model since the two profiles were very similar in shape and only slightly differed in levels of BAs. Therefore, based on these results, the four-profile solution was adopted as the preferred model. The BVR exceeding the value of 3.84 was noted between studying addiction and gaming addiction. The four-profile solution with the direct effect included had lower values of the model fit criteria than the four-profile solution without direct effects (see Table S2). Therefore, it was chosen as best-fitting the data.

Latent Classes (Students Positively Screened for at Least One BA)

The smallest values of BIC and CAIC were noted for a three-class solution. Values of the SABIC decreased with the addition of latent classes. The graphical representation of the SABIC showed the inflection of the curve around the third class (Figure S2). Moreover, the size of the smallest class in the four-class solution was as low as 4.6%. Thus, the three-class solution was adopted and used in further analyses. After inspecting the BVR values, two direct effects were introduced into the model (see Table S8).

Table S1. Comparisons Between Persons Without BAs and Persons with at Least One BA.

Variables	Persons Without BAs (n = 830)	Persons with at Least One BA (n = 327)	t-test/ χ^2 statistic [b]
	M (SD)	M (SD)	
Study addiction	15.05 (3.94)	21.11 (6.26)	-16.30 ***
Shopping addiction	10.81 (4.04)	14.83 (6.53)	-10.38 ***
Gaming addiction	11.71 (4.88)	13.93 (8.12)	-4.63 ***
Facebook addiction	11.08 (4.06)	14.85 (6.40)	-9.88 ***
Pornography addiction	8.13 (3.41)	9.95 (5.75)	-5.37 ***
Food addiction	15.99 (4.96)	22.67 (8.56)	-13.28 ***
Gender [a]	49.52%	58.10%	7.84 **
Age	20.24 (1.68)	20.55 (1.66)	-2.80 **
Extraversion	8.99 (2.86)	8.63 (2.99)	1.89
Agreeableness	9.85 (2.15)	9.31 (2.46)	3.51 ***
Conscientiousness	9.45 (2.56)	9.18 (2.89)	1.45
Emotional stability	8.79 (2.68)	7.71 (2.77)	6.11 ***
Openness	9.97 (2.21)	9.80 (2.29)	1.16
Narcissism	3.63 (2.01)	4.24 (2.38)	-4.06 ***
General quality of life	7.10 (1.33)	6.70 (1.48)	4.21 ***
Health quality	6.28 (2.00)	5.59 (2.20)	4.95 ***
Sleep quality	5.48 (2.09)	4.60 (2.20)	6.20 ***
Perceived stress	10.28 (2.81)	12.11 (2.95)	-9.61 ***
Anxiety	9.27 (2.71)	10.90 (3.06)	-8.41 ***
Hopelessness	8.46 (3.76)	10.35 (4.73)	-6.45 ***

Note. ** $p < 0.01$; *** $p < 0.001$. [a] For gender, the values present the percentage of women. [b] χ^2 statistic was only calculated for gender (0 = women, 1 = men). N = 1157.

Table S2. Comparison of LPA Models for Persons with at Least One Behavioral Addiction.

Model	LL	#p	BIC	CAIC	SABIC	Entropy
1-profile	-2780.95	12	5631.39	5643.39	5593.32	1.0
2-profile	-2328.82	25	4802.39	4827.39	4723.09	0.94
3-profile	-2199.30	38	4618.63	4656.63	4498.09	0.90
4-profile	-2066.49	51	4428.27	4479.27	4266.50	0.92
5-profile	-1986.67	64	4343.90	4407.90	4140.89	0.92
6-profile	-1928.18	77	4302.19	4379.19	4057.95	0.91
7-profile	-1840.97	90	4203.04	4293.04	3917.57	0.91
**4-profile***	**-2050.71**	**55**	**4419.86**	**4474.86**	**4245.40**	**0.90**

Note. LL = model log-likelihood; #p = number of parameters; BIC = Bayesian information criterion; CAIC = consistent AIC; SABIC: sample-size adjusted BIC. *Four-profile solution with a direct effect (between study addiction and gaming addiction) included. Bold values represent the best-fitting model. In this model, average posterior probabilities were 0.98, 0.90, 0.94, and 0.98 for Profiles 1, 2, 3, and 4, respectively. The expected misclassification rate was 4.49%. N = 327.

Table S3. Profile Comparisons for the Four-Profile Solution.

Potential BAs	Overall Wald test	Z-score for a Given Profile				Wald's Values for the Pairwise Comparisons among Profiles						Differences Between Profiles
		z_1	z_2	z_3	z_4	1 vs 2	1 vs 3	1 vs 4	2 vs 3	2 vs 4	3 vs 4	
Study addiction	14.09 **	0.26	-0.33	0.03	-0.09	**12.61**	1.59	**6.59**	3.09	2.04	0.48	1 > 2 = 4; 1 = 3; 2 = 3; 3 = 4
Shopping addiction	134.42 ***	-0.03	-0.76	0.00	0.56	**40.62**	0.05	**17.21**	**25.11**	**114.59**	**10.10**	4 > 1 = 3 > 2
Gaming addiction	383.58 ***	-0.84	0.68	0.07	0.47	**123.20**	**68.83**	**145.44**	**11.33**	1.32	**6.12**	2 = 4 > 3 > 1
Facebook addiction	184.96 ***	0.14	-0.86	-0.22	0.55	**71.47**	3.79	**9.44**	**14.38**	**164.26**	**17.48**	4 > 1 = 3 > 2
Pornography addiction	306.92 ***	-0.68	0.10	-0.68	1.09	**56.04**	0.02	**241.85**	**55.33**	**39.91**	**240.80**	4 > 2 > 1 = 3
Food addiction	139.04 ***	0.02	-0.84	0.03	0.52	**48.66**	0.00	**12.16**	**33.50**	**121.08**	**8.93**	4 > 1 = 3 > 2

Note. *$p < 0.05$; **$p < 0.01$; ***$p < 0.001$. BAs = behavioral addictions. All indicators were presented in a standardized form. For profile comparisons, a value of the Wald statistic higher than 3.84/6.63/10.83 is significant at p < 0.05/0.01/0.001, respectively. Bold values indicate the significant differences between the profiles. Profile 1: elevated levels of study, shopping, Facebook, and food addictions (33.7%); Profile 2: elevated levels of gaming and pornography addictions (20.1%); Profile 3: average or lower than average scores on all potential BAs (16.7%); Profile 4: elevated levels of most potential BAs (29.5%). N = 327.

Table S4. Prevalence and Co-Occurrence of Potential Behavioral Addictions (BAs) among Four Latent Profiles.

Potential BAs	Profile 1 (33.7%)	Profile 2 (20.1%)	Profile 3 (16.7%)	Profile 4 (29.5%)
Study addiction	62.8% (33.8%)	47.8% (9.1%)	51.9% (33.3%)	32.3% (73.3%)
Shopping addiction	12.4% (46.2%)	0% (0%)	11.5% (50.0%)	22.6% (81.0%)
Gaming addiction	0% (0%)	50.7% (8.6%)	9.6% (80.0%)	23.7% (63.6%)
Facebook addiction	22.1% (52.0%)	0% (0%)	17.3% (44.4%)	28.0% (76.9%)
Pornography addiction	0% (0%)	4.3% (0%)	0% (0%)	19.4% (72.2%)
Food addiction	34.5% (56.4%)	1.4% (0%)	40.4% (47.6%)	53.8% (64.0%)
Prevalence and co-occurrence of BAs	22.0% (31.4%)	17.4% (3.0%)	21.8% (42.6%)	30.0% (71.8%)

Note. The first value in each cell presents the percentage of members of a given profile who fulfilled the cutoff for a given BA. The values in parentheses present the percentage of members of a given profile who had at least one co-occurring BA. Profile 1: elevated levels of study, shopping, Facebook, and food addictions; Profile 2: elevated levels of gaming and pornography addictions; Profile 3: average or lower than average levels of all potential BAs; Profile 4: elevated levels of most potential BAs. N = 327.

Table S5. The Frequency of the Number of BAs among the Latent Profiles.

Number of BAs	Profile 1 (33.7%)	Profile 2 (20.1%)	Profile 3 (16.7%)	Profile 4 (29.5%)
One	74.3%	95.7%	73.1%	52.7%
Two	20.4%	4.3%	25.0%	30.1%
Three	4.4%	0%	1.9%	7.5%
Four	0.9%	0%	0%	5.4%
Five	0%	0%	0%	3.2%
Six	0%	0%	0%	1.1%

Note. Profile 1: elevated levels of study, shopping, Facebook, and food addictions; Profile 2: elevated levels of gaming and pornography addictions; Profile 3: average or lower than average levels of all potential BAs; Profile 4: elevated levels of most potential BAs.

Table S6. Summary Comparison of LCA Models for the Full Sample.

Model	LL	#p	BIC	CAIC	SABIC	Entropy
1-profile	-1592.37	6	3227.06	3233.06	3208.00	1.0
2-profile	**-1517.83**	**13**	**3127.35**	**3140.35**	**3086.06**	**0.55**
3-profile	-1505.74	20	3152.54	3172.54	3089.02	0.39
4-profile	-1503.46	27	3197.37	3224.37	3111.61	0.36
5-profile	-1502.07	34	3243.97	3277.97	3135.97	0.33
6-profile	-1501.18	41	3291.57	3332.57	3161.34	0.40
7-profile	-1500.78	48	3340.12	3388.12	3187.66	0.35

Note. LL = model log-likelihood; #p = number of parameters; BIC = Bayesian information criterion; CAIC = consistent AIC; SABIC: sample-size adjusted BIC. Bold values represent the best-fitting model. Average posterior probabilities for the final (i.e., two-class) model were 0.96 for Class 1 and 0.80 for Class 2. The expected misclassification rate was 4.64%. N = 1157.

Table S7. Class Comparisons for the Two-Class Solution.

Potential Behavioral Addictions	Conditional Probabilities		Comparison of Latent Classes (Wald test)
	p_1	p_2	1 vs 2
Study addiction	0.11	0.45	**37.36**
Shopping addiction	0.02	0.23	**38.43**
Gaming addiction	0.04	0.16	**12.64**
Facebook addiction	0.02	0.34	**52.22**
Pornography addiction	0.01	0.11	**20.01**
Food addiction	0.01	0.66	**51.89**

Note. Bold values indicate the significant differences between the classes ($p < 0.001$). Class 1 (91.0%): a very low probability of potential BAs; Class 2 (9.0%): the heightened probability of all BAs.

Table S8. Comparison of LCA Models for Persons with at Least One Behavioral Addiction.

Model	LL	#p	BIC	CAIC	SABIC	Entropy
1-profile	-952.20	6	1939.14	1945.14	1920.11	1.0
2-profile	-906.42	13	1888.10	1901.10	1846.86	0.73
3-profile	-871.10	20	1858.00	1878.00	1794.56	0.79
4-profile	-857.49	27	1871.31	1898.31	1785.67	0.91
5-profile	-840.15	34	1877.17	1911.17	1769.32	0.94
6-profile	-825.55	41	1888.48	1929.48	1758.43	0.92
7-profile	-812.49	48	1902.89	1950.89	1750.64	0.92
**3-profile***	**-863.46**	**22**	**1854.30**	**1876.30**	**1784.52**	**0.82**

Note. LL = model log-likelihood; #p = number of parameters; BIC = Bayesian information criterion; CAIC = consistent AIC; SABIC: sample-size adjusted BIC. *Three-class solution with two direct effects included (between gaming addiction and shopping addiction, and Facebook addiction and food addiction). Bold values represent the best-fitting model. In this model, average posterior probabilities were 0.91, 0.93, and 0.97 for Classes 1, 2, and 3, respectively. The expected misclassification rate was 7.28%. N = 327.

Table S9. Class Comparisons for the Three-Class Solution.

Potential BAs	Overall Wald test	Conditional Probabilities			Wald's Values for the Pairwise Comparisons among Classes			Differences Between Classes
		p_1	p_2	p_3	1 vs 2	1 vs 3	2 vs 3	
Study addiction	11.91 **	0.25	1.00	0.10	**8.87**	2.48	**11.09**	2 > 1 = 3
Shopping addiction	9.38 **	0.22	0.03	0.00	**7.73**	1.69	0.77	1 > 2; 1 = 3; 2 = 3
Gaming addiction	15.89 ***	0.10	0.00	0.99	0.57	**15.22**	**5.89**	3 > 1 = 2
Facebook addiction	10.24 **	0.35	0.03	0.00	**7.93**	2.56	0.51	1 > 2; 1 = 3; 2 = 3
Pornography addiction	4.65	0.12	0.01	0.00	**4.02**	0.66	0.20	1 > 2; 1 = 3; 2 = 3
Food addiction	25.20 ***	0.61	0.09	0.03	**20.55**	**6.95**	0.49	1 > 2 = 3

Note. ***p* < 0.01; ****p* < 0.001. All indicators were presented in a standardized form. For class comparisons, a value of the Wald statistic higher than 3.84/6.63/10.83 is significant at *p* < 0.05/0.01/0.001, respectively. Bolded values indicate the significant differences between the classes. Class 1: the heightened probability of almost all potential BAs (50.4%); Class 2: a very high probability of study addiction and a very low probability of other BAs (35.5%); Class 3: a very high probability of gaming addiction and a very low probability of other BAs (14.1%). N = 327.

Table S10. The Accuracy of Classification of Cases in General Student Sample and Subsample Positively Screened for BAs Based on LPA.

				General Student Sample							
				General proclivity		Female-majority		Male-majority		Average or low levels of BAs	
				Number (percentage) of all cases							
				259 (22%)		336 (29%)		288 (25%)		274 (24%)	
				Cases that entered into subsample							
				141 (54%)		102 (30%)		37 (13%)		47 (17%)	
		n	%[a]	N	%	n	%	n	%	n	%
Subsample positively screened for at least on BA	General proclivity	93	8%	**93**<br>**66%** [c]	**100%** [b]						
	Female-majority	113	10%	8<br>6% [c]	7% [b]	**102**<br>**100%** [c]	**90%** [b]			3<br>6% [b]	3% [b]
	Male-majority	69	6%	21<br>15% [c]	30% [b]			**37**<br>**100%** [c]	**54%** [b]	11<br>23% [c]	16% [b]
	Average or low levels of BAs	52	5%	19<br>13% [c]	37% [b]					**33**<br>**70%** [c]	**63%** [b]

Note. Zero frequencies and percentages are not shown for clarity reasons. [a] Percentage of the general student sample. [b] Percentage of raw (profile in subsample). [c] Percentage of cases from particular profile in general student sample classified in the subsample. Bold values represent percentages in the corresponding profiles in the full sample and subsample.

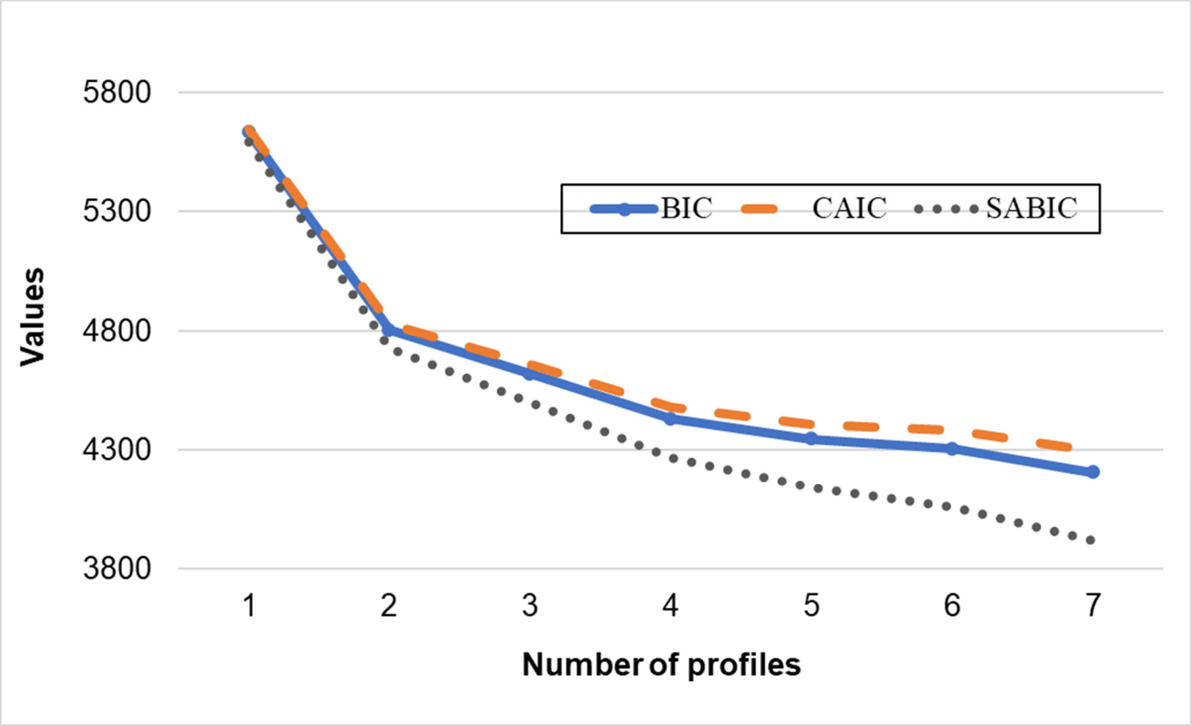

Figure S1. Elbow Plot of the BIC, CAIC, and SABIC Values for the LPA Solutions. N = 327.

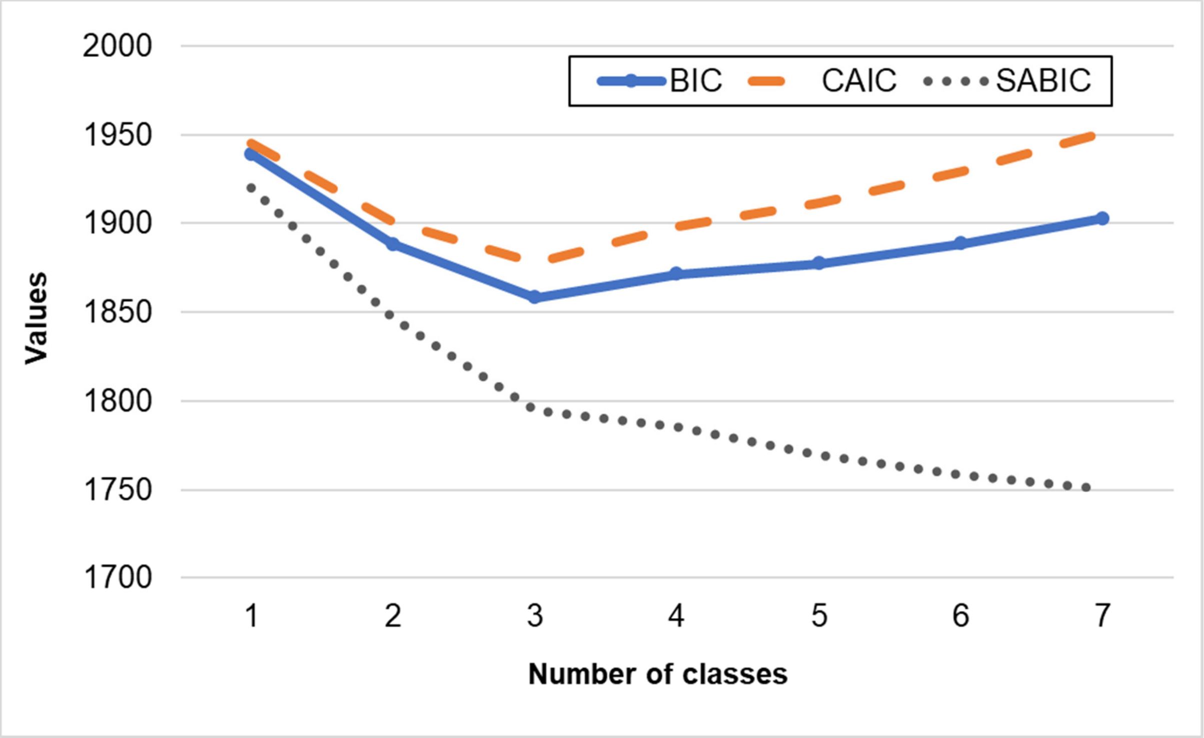

Figure S2. Elbow Plot of the BIC, CAIC, and SABIC Values for the LCA Solutions. N = 327.
